# Pregnancy-specific glycoproteins as molecular links between mesenchymal stromal cells and M2 macrophages in tissue repair: implications for cancer progression

**DOI:** 10.3389/fcell.2026.1798621

**Published:** 2026-03-18

**Authors:** Lindolfo da Silva Meirelles

**Affiliations:** Lutheran University of Brazil, Canoas, Brazil

**Keywords:** cancer, cancer-associated fibroblasts, M2 macrophages, mesenchymal stromal cells, MSCs, pericytes, pregnancy-specific glycoproteins, wound healing

## Abstract

After some decades of research on mesenchymal stromal cells (MSCs), they are not yet routinely used in clinical protocols. Even though various lines of investigation have pointed out that MSCs are therapeutic mainly owing to their secreted molecules, and a number of such molecules have been described, it is apparent that further understanding of MSC biology is still required to make them effective tools to treat diseases. This mini-review brings the perspective that MSCs do not act alone, but rather in concert with other cell types such as macrophages, to bring about tissue regeneration or, in some instances, unwanted consequences. In this context, the possible effects of pregnancy-specific glycoproteins produced by MSCs on macrophages, and consequences of this for tissue repair, cancer progression, and chimeric antigen receptor T cell therapy in solid tumors will be discussed.

## 1 MSCs

Mesenchymal stromal cells (MSCs) show promise as tools to treat a wide range of conditions. A search on www.clinicaltrials.gov performed in January 2026 using the search term [“mesenchymal stromal cells” OR “mesenchymal stem cells” OR “MSC”] in the “intervention/treatment” field returned 1,853 studies. Of these, 212 were recruiting patients, while 124 were not yet recruiting, and 67 were active but not recruiting; whereas, 638 trials were completed, and 76 were terminated. The conditions to which mesenchymal stromal cells (MSCs) were administered included COVID-19, graft-versus-host disease, Crohn’s disease, multiple sclerosis, osteoarthritis, ischemic stroke, sepsis, spinal cord injury, and organ transplantation, among many others. In spite of the numerous clinical trials using MSCs, their use in routine clinical protocols is yet not a reality in humans.

Even though MSCs were originally described primarily as fibroblastic cells that could form colonies when cultured (Friedenstein et al., 1970; 1974), they were later found to have osteogenic and chondrogenic potential (Friedenstein et al., 1987). Eventually, MSCs became regarded by a portion of the scientific community as mesenchymal stem cells owing to their ability to acquire characteristics of cells from bone, cartilage, and adipose tissue when cultured under appropriate conditions (Caplan, 1991; Pittenger et al., 1999), while others proposed that these cells should be called mesenchymal stromal cells (Horwitz et al., 2005). The differentiation abilities of MSCs prompted for their use in tissue engineering (Caplan and Bruder, 2001). The assumption that cultured MSCs would home to mesenchymal tissues and differentiate into parenchymal cells when infused in the body led to the expectation of their use in cell replacement therapies for various conditions including osteogenesis imperfecta, for example (Caplan, 1995).

After apparent success in preclinical models of osteogenesis imperfecta (Pereira et al., 1995; 1998), MSCs were ultimately found to be incapable of long-term engraftment when administered to patients affected by this or other conditions in spite of improvements observed after the treatments (Horwitz and Dominici, 2008). Indeed, experiments in animals showed that MSC administration elicits improvement after tissue damage, but not by means of differentiation (Tang et al., 2004; 2005; Horwitz and Dominici, 2008). Such findings led to the suggestion that MSCs could be therapeutic not necessarily by differentiating into mature tissue cells, but by secreting signaling molecules with trophic and immunomodulatory effects (Caplan and Dennis, 2006). Meanwhile, researchers began to find evidence that extracellular vesicles produced by MSCs contained molecules that could help heal injuries (Bruno et al., 2009), which attested this mechanism of action.

MSCs can be obtained from virtually any vascularized tissue, and are absent in circulating blood under normal conditions (da Silva Meirelles et al., 2006). The presence of MSCs throughout the body has been attributed to their identity as a proliferative form of pericytes, cells that surround endothelial cells in the blood vessels under steady-state conditions (Bianco and Cossu, 1999; Crisan et al., 2008). These findings eventually led to the suggestion that tissue injury prompts local activation of pericytes that, in this activated state, become the *in vivo* equivalent of cultured MSCs and secrete trophic and immunomodulatory molecules that promote tissue healing by reducing inflammation and stimulating tissue-intrinsic progenitors (da Silva Meirelles et al., 2008).

The idea that pericytes can give rise to MSCs after injury in conjunction with additional earlier studies indicating that pericytes can give rise to mature cells under injury conditions has led to the suggestion that pericytes could be mesenchymal stem cells (Caplan, 2008; 2009). While a number of lineage tracing studies found evidence that pericytes give rise to differentiated progeny *in vivo*, evidence on the contrary also emerged (Da Silva Meirelles et al., 2024). While studying this subject, our group compared the transcriptomes of cultured MSCs, cultured pericytes, and freshly isolated, non-cultured pericytes from human adipose tissue (da Silva Meirelles et al., 2015; 2016). In those studies, the gene expression profile of pericytes cultured under standard MSC conditions was found to be nearly identical to that of MSCs obtained through traditional methods (da Silva Meirelles et al., 2015). Additionally, non-cultured pericytes were found to express message for markers associated with adipose tissue stem or progenitor cells identified in previous lineage tracing studies (da Silva Meirelles et al., 2016). When non-cultured pericytes were compared to MSCs by means of differential expression analysis, another pattern became apparent: transcripts encoding cytokines chemoattractant to inflammatory cells, namely *CXCL8*, *CXCL3*, *CXCL2*, and *CXCL1* were among the top 15 transcripts most differentially expressed by non-cultured pericytes; notably, transcripts coding for pro-inflammatory molecules were not represented among those differentially expressed by MSCs as compared to non-cultured pericytes (da Silva Meirelles et al., 2016). These results suggest that non-cultured pericytes, whose isolation process inevitably mimics some aspects of early tissue injury, have a pro-inflammatory phenotype while MSCs, whether obtained by traditional methods or by culture of pericytes under MSC conditions, have an anti-inflammatory profile.

Traditionally, studies on the immunosuppressive effects of MSCs have been focused on lymphocytes, with various research groups showing that MSCs regulate the activity of lymphoid cells by secretion of molecules such as transforming growth factor beta (TGFβ), prostaglandin E2 (PGE2), interleukin (IL) 10 (IL-10), leukemia inhibitory factor (LIF), by conversion of tryptophane into L-kynurenine by 2,3-dioxygenase, and by direct physical contact (Meirelles et al., 2009). Conversely, studies on the paracrine effects of MSCs on innate immune cells indicate they regulate macrophage phenotype by secretion of molecules such as TGF-β (Liu et al., 2019) and PGE2 in addition to physical contact (Chiossone et al., 2016). It is in this context that the abovementioned study on the gene expression of non-cultured pericytes and MSCs provides additional insight. In that study, two transcripts, namely *PSG3* and *PSG8*, were among the top eight differentially expressed in cultured MSCs as compared to non-cultured pericytes. *PSG3* and *PSG8* stand for pregnancy-specific glycoprotein three and pregnancy-specific glycoprotein 8, respectively. The expression level of these transcripts was virtually nil in non-cultured pericytes and high in cultured MSCs, making them 820-fold (*PSG3*) and 299-fold (*PSG8*) more expressed in the latter (da Silva Meirelles et al., 2016). At first, this observation did not seem to be important; however, a closer look at these transcripts could prove worthwhile.

## 2 PSGs

Pregnancy-specific glycoproteins (PSGs) are a family of molecules produced by placental trophoblasts and secreted into the maternal bloodstream; PSGs belong to the immunoglobulin superfamily and are closely related to carcinoembryonic antigen-related cell adhesion molecules (CEACAMs) (Moore et al., 2022). The first description of PSGs dates back to the 1970s, with the first of them described as a pregnancy-specific glycoprotein with the electrophoretic mobility of a β_1_ globulin, hence falling under the early acronym PSβ_1_G (New placental proteins, 1977). In humans, the PSG gene family comprises nine genes (*PSG1* - *PSG9*, and *PSG11*) and one expressed pseudogene (*PSG10P*) (Moore and Dveksler, 2014). *PSG7* is peculiar in the sense that nearly 70% of the population is expected to be homozygous for a single nucleotide polymorphism that creates a stop codon in exon 2, which possibly results in a truncated, short non-functional protein (Moore et al., 2022).

## 3 The effects of PSGs on macrophage phenotype

In search for specific functions of PSβ_1_G, some research groups tested the hypothesis that it could be involved in immunoregulation in the mother’s body through pregnancy. In 1977, suppression of phytohemagglutinin-induced T cell activation *in vitro* by PSβ_1_G was reported (Cerni et al., 1977). Later, PSβ_1_G was found to inhibit lymphocyte activation triggered by phytohemagglutinin but not by pokeweed mitogen, and to inhibit lymphocyte activation at physiological concentrations more potently in a mixed lymphocyte reaction, a condition that better simulates an *in vivo* scenario (Harris et al., 1984). In that study, while discussing whether PSβ_1_G could act directly on T cells, the authors mentioned that they had “…not, however, excluded an effect of accessory cells.” Such a statement was fortunate: nearly 15 years later, it was established that recombinant PSG1, PSG6, or PSG11 behave as immunomodulatory molecules by inducing secretion of IL-10, IL-6, and transforming growth factor β1 (TGFβ1) in human monocytes (Snyder et al., 2001), after the demonstration, in mice, that PSG18 induces IL-10 production in murine macrophages (Wessells et al., 2000). Soon, researchers demonstrated that recombinant PSG1a can induce alternative activation of monocytes in mice, as shown by upregulation of arginase and downregulation of nitric oxide production in lipopolysaccharide-activated monocytes (Motrán et al., 2002).

The macrophage is one of the main players in the inflammation that ensues early after tissue injury, along with neutrophils (Eming et al., 2017). Under steady-state conditions, tissue-resident macrophages play an important role in physiological tissue repair and maintenance (Guan et al., 2025). Experimental evidence indicates that acute inflammation leads to loss of most local tissue-resident macrophages in a matter of hours after an insult, which coincides with the ingress of neutrophils into the tissue (Melnicoff et al., 1989) – this phenomenon has been called “macrophage disappearance reaction” (Barth et al., 1995). In spite of that, during the initial stages of the response to an injury, damage-associated molecular patterns (DAMPS) and/or pathogen-associated molecular patterns (PAMPs) instigate not only surviving tissue-resident macrophages, but mainly macrophages that develop from infiltrating monocytes, to become activated and take up a pro-inflammatory phenotype; these classically activated macrophages are called M1 macrophages (Mills, 2012). As the wound healing process progresses, alternatively activated, pro-regenerative macrophages become more prevalent; these macrophages, collectively called M2 macrophages, reduce inflammation, promote extracellular matrix production, and stimulate angiogenesis by secreting molecules like TGFβ, IL-10, and vascular-endothelial growth factor (Hesketh et al., 2017). It is noteworthy that the classification of macrophage phenotypes into M1 and M2 is an oversimplification of a range of possible phenotypes, as various subtypes of M1 and M2 macrophages have been defined *in vitro*, and a continuum of macrophage phenotypes is expected to exist *in vivo* (Yan et al., 2024). With that in mind, in this mini-review, the M1/M2 macrophage dichotomy is used only for the sake of simplification.

In mice, binding of PSG19 to its receptor, CD9, on macrophages induces secretion of IL-10, IL-6, PGE2, and TGFβ1 (Ha et al., 2005), which are characteristic of various macrophage phenotypes of the M2 spectrum (Strizova et al., 2023). In addition to inducing secretion of TGFβ by macrophages, PSGs can also determine activation of the latent form of this cytokine by physically interacting with it, as demonstrated for PSG1 (Blois et al., 2014) and PSG9 (Jones et al., 2016). Blois et al. (2014) further demonstrated that administration of PSG1 protects mice against experimentally induced colitis, with consequent decrease in levels of the pro-inflammatory cytokines interferon gamma, tumor necrosis factor, and IL-17, and an increase in the level of the anti-inflammatory cytokine IL-10 (Blois et al., 2014). Together, the studies above show that PSGs have an important effect on the polarization of macrophages toward an M2 phenotype not only by triggering the production of anti-inflammatory molecules including TGFβ, but also by rendering the latter biologically active.

## 4 The interplay between MSCs, fibroblasts, PSGs, and M2 macrophages

**FIGURE 1:**
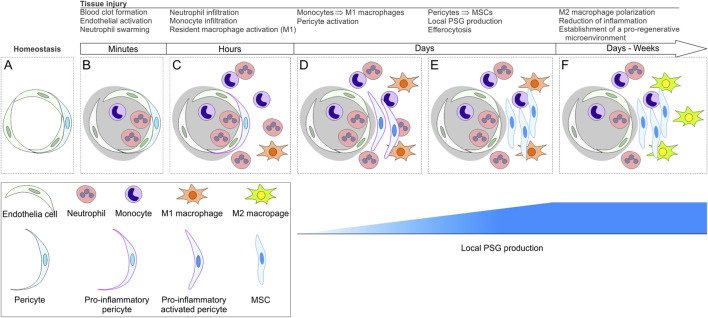
Schematic representation of cellular and molecular events that take place after tissue injury. For sake of simplification, various cell types, including parenchymal cells, are not represented. **(A)** representation of a transversal section of a small blood vessel. In **(B)** an injury event leads to local tissue damage and formation of a blood clot (gray area). The endothelium becomes activated, and neutrophils accumulate at the injured site, along with monocytes. In **(C)** pericytes assume a pro-inflammatory phenotype. Neutrophils and monocytes ingress into the damaged parenchyma. Surviving local macrophages take up a classically activated phenotype (M1). In **(D)** monocytes differentiate into macrophages that become pro-inflammatory (M1). Whereas, pericytes become activated, proliferate, and begin to undergo phenotypic changes. Tissue infiltration of neutrophils and monocytes continues. **(E)** gene expression changes that began in D lead to the conversion of pericytes into mesenchymal stromal cells (MSCs), which secrete pregnancy-specific glycoproteins (PSGs). Macrophages phagocytose dying neutrophils (efferocytosis). In **(F)** pro-regenerative (M2) macrophages become prevalent. A pro-regenerative microenvironment is established; the ingress of neutrophils and monocytes decreases.

**FIGURE 2:**
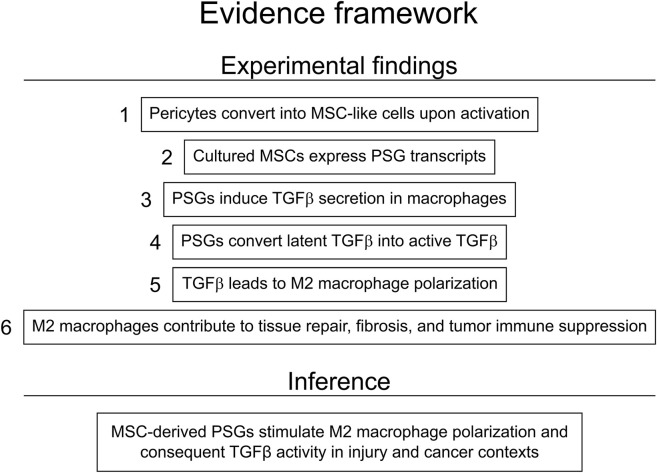
Evidence framework for a proposed mechanism of M2 macrophage polarization induced by pregnancy-specific glycoproteins produced by mesenchymal stromal cells (MSCs). Experimental findings one and six have been demonstrated *in vitro* and *in vivo*. Experimental findings two to five have been observed *in vitro*.

Our group has previously proposed that MSCs, whether arising locally from pericytes or obtained through cell culture, play a major role in the tissue repair by promoting the development of pro-regenerative macrophages, which may outlive MSCs as the wound healing process progresses (da Silva Meirelles et al., 2020; Marson et al., 2023) In this context, the finding that MSCs express high levels of PSGs (while non-cultured pericytes express none) becomes relevant as these molecules are expected to trigger the production of TGFβ in local macrophages after tissue injury, potentiating the stimuli for M2 polarization and consequent generation of a pro-regenerative microenvironment by M2 macrophages (Figures 1, 2). Clearly, it is possible that other cells involved in this process could produce PSGs too, as it has been demonstrated that endothelial-to-mesenchymal transition (EndMT) can give rise to cells with MSC characteristics under injury conditions *in vivo* (Medici et al., 2010). EndMT has been shown to be inducible by TGFβ and to contribute to the development of fibroblasts in fibrosis (Zeisberg et al., 2007b; 2008) and cancer (Zeisberg et al., 2007a). In this scenario, PSGs produced by stromal cells originating from pericytes after an injury event could instigate macrophages to produce TGFβ, which, in turn, favors EndMT and consequent production of fibroblasts. Even though EndMT may play a role in the development of fibroblasts, it is important to note that pericytes have been shown to be the main source of fibroblasts in various tissues *in vivo* (Humphreys et al., 2010; Kramann et al., 2015; Dias et al., 2021), and that the production of PSGs by fibroblasts (Rosen et al., 1979; Heikinheimo et al., 1981; Chou et al., 1989), particularly when they reach replicative senescence (Endoh et al., 2009), has been reported. Looking back to their first accounts as fibroblastic cells (Friedenstein et al., 1970; 1974), differences between MSCs and fibroblasts remain difficult to tell (Haniffa et al., 2009; Hematti, 2012). As a word of caution, it must be noted that the mechanism proposed in this section remains, at this point, rather inferential and warrants further demonstration of PSG production by fibroblasts/MSCs *in situ* during tissue repair.

## 5 PSGs and stromal cells in cancer

While the production of PSGs by fibroblastic stromal cells that develop after tissue damage could be important for the establishment of regenerative microenvironment rich in M2 macrophages and consequent wound healing, it is possible that this process might have some unwanted consequences in some instances, particularly in cancer. Tumors have been suggested to be wounds that do not heal, in which tumoral cells exploit aspects of the traditional wound healing process “…as a means to acquire the stroma they need to grow and expand” (Dvorak, 1986). Under this perspective, local PSG production by MSCs/fibroblasts that arise following an injury event, or events that mimic aspects of injury, could promote M2 macrophage polarization with consequent production of high amounts of TGFβ. In experimentally induced liver cancer following chronic liver injury, genetic lineage tracing indicated that liver-specific pericytes (hepatic stellate cells) became activated and gave rise to around 65% of all myofibroblasts in fibrotic livers, and nearly 85% of cancer-associated fibroblasts (CAFs) (Wang et al., 2021); in that study, conditional deletion of the TGFβ receptor Tgfbr2 resulted in decreased hepatic fibrosis and a smaller tumor area, along with increased numbers of CD8^+^ T cells.

CAFs are the most abundant stromal cells of the tumor microenvironment (TME), and have been implicated in immunosuppression, M2 macrophage polarization, and CD8^+^ T cell exclusion through mechanisms that include TGFβ secretion (Lan et al., 2025). Administration of anti-TGFβ antibodies to a mouse model has resulted in some improvement on the outcome of a treatment using a monoclonal antibody against cancer cells positive for PD-L1, a molecule that precludes CD8^+^ T cells from performing their anti-tumoral cytotoxicity (Mariathasan et al., 2018). However, the immunosuppressive nature of the TME poses a formidable challenge even for high-end cancer treatments such as those using chimeric antigen receptor (CAR)-T cells, since this microenvironment recruits regulatory T cells, myeloid-derived suppressor cells, and tumor-associated macrophages that work in concert to suppress cytotoxic T cell activity; consequently, CAR-T cell therapy remains ineffective against solid tumors (Kong et al., 2024). In this context, PSGs could represent a novel molecular target upstream of TGFβ in the treatment of solid tumors, particularly when associated with CAR-T cell therapy. At the moment, the assumption that PSGs produced by tumoral stromal cells facilitate cancer progression by favoring M2 macrophage polarization is inferential (Figure 2) and has yet to be verified. Testable predictions of this hypothesis include a positive correlation between PSG-rich stromal niches and increased frequency of M2 macrophage markers along with active TGFβ signatures, and enhanced T cell infiltration or CAR-T cell persistence after PSG blockade in preclinical models.

## 6 Outlook

For a considerable time, MSCs have been deemed therapeutic as standalone tools to treat various conditions. The current state of the knowledge on MSC biology suggests that they do not act alone to promote tissue repair but, instead, help establish a microenvironment that instigates the development of M2 macrophages that carry on the regenerative process. PSGs appear to be overlooked as MSC-secreted molecules that integrate this complex system. Further research on PSGs could not only advance the understanding of the events involved in wound healing, but also provide opportunities for the development of novel approaches to treat a wide range of diseases, including cancer.
